# Editorial Department

**Published:** 1883-10

**Authors:** 


### The Bistoury.

ELMIRA, N. Y., OCTOBER, 1883.

The BISTOURY is published Quarterly, upon the first of April, July, October and January, at Fifty Cents a year in advance.

### Editorial Department.

THE OPERA HOUSE.—Our only recognized place of amusement in this city has become so outrageously filthy as to bar decent people—those having regard for their surroundings—from going there. It would seem that the city has attained such proportions as to warrant capitalists in erecting a suitable place of amusement for her citizens. Let it be upon the ground floor, too, with abundant opportunities for ingress and egress. The present place is dangerous and dirty.

FRINGED GENTIAN, the loveliest of all wild flowers, are now (Sept. 20th) in bloom. Search in the moist spots on the hill-tops for them. The ramble will be invigorating, and a bunch of this beautiful and rare flower most rewarding. As we write, a cluster of them brightens up our window-sill, reminding us of the self-sacrificing little woman who carried them to us, accepting as ample pay, our enthusiastic admiration of them. Place them in a vase of warm water, to which has been added a teaspoonful of aqua-ammonia, sit them in a cool place, when they will bloom for many days, greatly to your delight and for your admiration.

SECRETARY OF STATE.—The political organization known as the Greenback Party are proud of the enlistment in their cause of so able and popular a gentleman as Thomas K. Beecher, so they nominate him whenever opportunity offers, for some office. Once, they came within a very few hundred votes of electing him to Congress, he having polled the largest vote ever cast in this county for any candidate. Now, they name him as their candidate for Secretary of State. Of course, they cannot elect him,—the more's the pity, for Mr. Beecher certainly has all the qualifications necessary to make a most excellent officer. No one will doubt his honesty, integrity and great capability for transacting the business of the office for which he has been nominated. No better designation has been made, by either of the great parties, of a suitable person to fill the chair of Secretary of State. We would be glad to see him elected, no matter whether the Greenback ticket succeeds or not.

DRS. BLACKHAM AND DUNNING ON CARLYLE.—It will be seen that Dr. Blackham criticises Carlyle quite severely; and, as many will adjudge not undeservedly, in the present number of THE BISTOURY. Dr. Dunning, an inmate of the Institute, seeing Dr, Blackham's article in proof, and being an ardent admirer of Carlyle's writings, asked and obtained the privilege of a reply in the same number. Dr. Blackham wrote, in a private communication to the editor: "Give Dr. Dunning a show. If any one can write anything in favor of Carlyle, it ought to be done." So we have the two excellent articles, *pro* and *con*, in this number.

The same may be said of Dr. Corey's contribution, which is replied to by Dr. Carr. The reply was written in THE BISTOURY office, after Dr. Corey's article was in proof.

BACTERIA AND DR. GREGG.—We see it announced in the Buffalo papers that Dr. Gregg [likely, Dr. Rollin R. Gregg, a very capable microscopist] has asserted that bacteria are *not* the cause of certain diseases to which they have heretofore been ascribed; that bacteria are *always* to be found present in the blood, saliva and other fluids of the body, and that therefore their presence is not indicative nor provocative of disease; and, further, that the so-called bacteria are not living organisms at all, but simply particles of fibrine.

We have not seen Dr. Gregg's paper, nor even an intelligent synopsis of it; so, cannot determine how correctly he may be reported in the daily papers referred to. Of this, however, we may be assured, that it will require very strong evidence, supported by practical experiments, to disprove the theories set up by Pasteur, Koch, and other capable experimenters with bacteria and disease.

All that is known of bacteria, has by no means exhausted the opportunities for experimentation and research among them. There exists here, ample means for investigation, and we should carefully consider all that is written upon the subject, when it comes from a capable and careful microscopist like Dr. Gregg. Because a man "diverges from the generally accepted theories of scientists," is no reason for supposing him to be wrong. Contrariwise, it is all the more possible that he is right. Do not condemn an investigator for advancing a new theory—differing from the accepted one—because he *may* prove his position some day, by incontrovertible facts, much to your dismay and chagrin.

---

THE STRATFORD CASE.—Much interest clings to the mysterious murder of Rose Ambler. Notwithstanding many expert detectives have been trying to discover the parties concerned in her murder, no evidence of sufficient importance has yet been found to implicate any one in the crime. Here, again, was the microscope used as a detective. Under the finger nails of the murdered girl were found quantities of epithelial scales scratched, presumedly, from the face and hands of her slayer, in her frantic attempts to release herself from his grasp. These scales were subjected to inspection under the microscope and proved to be from the skin of a white, adult, sun-burned person. No epithelial scales from the person of a negro were found, showing that the individual with whom she had her death struggle was not a black man, as was at first supposed.

And so, every year, as its capabilities become better known, the value ofthe microscope, as a detective in many criminal cases, is readily recognized.

---

ANOTHER DEATH.—It will be noticed that our contributor, Mr. J. B. Chandler, refers to the death of a very dear friend. We were fortunate in an intimate acquaintance with the person referred to—Mr. Wm. C. Richardson, of Philadelphia, a lover of nature and of the gentle art of angling. Often have we met him upon the mountain streams in the Alleghanies, and, seating ourself by his side upon a rock in the stream, or on a mossy bed of violets upon the bank, hear him deliver a eulogy upon the delightful occupation of angling. He thoroughly enjoyed the mountain scenery—the woods—birds—flowers—and all the joyful surroundings that made the following of the angler's art so attractive to a cultured mind like his. Never very robust, he was often found alone, seated in some mossy, crystal dell, drinking in the pure mountain air, enjoying the musings of his own mind upon the beauties of nature here so lavishly displayed. He was a skillful angler, a cultured and refined gentleman, a true friend, and an honest man. May the rewards that should accompany these noble attributes be his.

---

SUICIDE.—Our readers have scarcely failed to have noticed the exceeding prevalence of suicide that has taken hold upon all classes of people. It has become an unusual occurrence to peruse the morning paper without encountering the particulars of a self-murder, that day, somewhere imposed.

The right and wrong of suicide has been extensively discussed recently, because of the prominence given the subject through the writings of a noted pastor of this city, who thinks man, in many instances, excusable for laying down his life. This is such an unusual attitude for a minister of the church to sustain towards that question that innumerable daily papers have thought best to chastise him with words—simply words—for attempting to extenuate in any manner, what *they* were taught to believe a great crime.

Our sympathies go with the Pastor. He is right; and, we doubt whether any intelligent person will sincerely dispute his premises. See:—Given, a person who is suffering, and has *long* suffered, from cancer of the stomach. His physicians say he is incurable. He must die, eventually, from the terrible ailment. The patient recognizes the fact, as does his family, and all are waiting for the day to come that shall give the sufferer rest. Meanwhile, he suffers untold agony, daily, hourly; prays for death to come, that he may be delivered from his torture, while his friends hope and pray for a quick deliverance for him. Will any thoughtful person say it is wrong for that person to take two grains of morphine, subcutaneously?—to commit suicide? We think not. There may be many situations equally well-defined, in which man would be justifiable in taking his own life—particularly where others are benefited as well as himself by the "chivalrous sacrifice."

The only wonder to us is that so many persons cling to painful and distressing lives, that are really not worth the having, and the great hardship in the possession of which, is in no manner compensated for by the slender pleasure of simply breathing. We fail to see the benefit to be derived—save, perhaps, in special instances—from prolonging the life of one who is anxious to have it cease, that he may be released from the suffering of an incurable disease, his family spared the distress of witnessing his suffering—the expense of suitably caring for him, and, perhaps, prevented from earning bread for the remainder of the household, because of duty and service due the sufferer. We cannot adduce any tenable argument prevailing upon such a person to try to live. He should be permitted to die if he chooses, and no one should throw any obstacle in his way. Let him commit suicide.

---

CREMATION, AGAIN.—Our readers are aware of the industry with which we have urged, heretofore, the burning of dead bodies, instead of their inhumation. Added importance is given the theme since the recommendation of the Grand Jury of New Orleans, that a crematory be at once established in that city, simply upon sanitary grounds, to the end that all bodies of those who die of contagious diseases may be disposed of in such a manner as not to endanger the lives of the living. Dr. Domingo Freire, of Rio de Janeiro, has been investigating the cause of yellow fever, and found that *one* of the reasons of its prevalence in that city was to be attributed to the cemeteries where the victims of the disease were buried. The soil of these depositories of the dead was found to be alive with the micro-organisms supposed to be responsible in producing that dreaded disease. It has already been shown that typhoid, scarlet, and other infectious and contagious fevers have been propagated from wells and water courses lying in proximity to cemeteries, so that the danger in spreading disease from buried dead bodies is well understood. Intelligent people recognize this danger, and are only prevented from adopting the more healthful and cleanly method of disposing of the dead through a mere matter of sentiment or regard for ancient religious ceremonies.

To those who look upon cremation with repugnance, another method is offered, quite as effectual as cremation, being, in fact, only another way of burning the body and preventing decomposition, while the germs of disease are probably quite as effectually destroyed. This may be accomplished by placing tbe body in a willow basket, woven in the form of the usual casket, and, after it is deposited in the grave, a barrel of quick lime can be deposited thereon, the grave filled up, and the body left to be consumed by chemical action of the slackening lime. This process might not be so repulsive to some sensitive natures as the quicker burning by fire, and will meet the requirements of sanitary precaution quite as well. We commend the process to those who are willing to aid in bringing about an improved method in burying the dead, and so lessening the number of dangers that surround the living.

---

CHECKS ON DISHONESTY.—We listened, a few evenings ago, to a very interesting lecture by Mr. Beecher, upon the above subject, in which he referred to the manifold contrivances that are employed by men, designed to prevent their cheating one another.

The duplex-ticket, bell punch, indicators and "spotters" of the steam and horse railways were commented upon as being debasing and humiliating to the men compelled to employ them. It was argued that the very means used as preventives of dishonesty of the employed against the employer, acted as a constant reminder to the former of the suspicion cast upon him, souring his disposition and giving rise to feelings and desires for revenge and for a means to circumvent the ingenious contrivances of the employer.

That argument is doubtless true; but, human nature being what it is, something of the sort seems necessary for the protection of the employer.

Recently, we were the owner of a large red cow. She was a restless, vicious sort of a beast, that enjoyed nothing better than to jump over, or creep under, the fence that surrounded her excellent pasture, that she might run over or lie down in a neighboring garden. The owner of the garden remonstrated with her without inducing her to desist; so, a bill of damages was presented to her owner, which was promptly paid. Soon, another bill, of similar amount, for the nefarious practices of that abominable lacteal purveyor, in the same garden, was presented. This sum was also paid, making the charges against the red cow an even ten dollars. The owner then thought best to either purchase that garden or sell the cow; so he sallied forth on a tour of inspection. He found the garden—it was planted with two rows of corn, just five feet long, and with potatoes, for ten square feet! Upon this patch he had paid damages amounting to ten dollars for the frolics of his red cow. Now, did not the owner of that patch need a bell-punch or a "spotter," of some sort? Well, we concluded to sell the cow, with the hope of avoiding further claims for damages; for, a chap hearing of the success of his neighbor in collecting "damages" from the fence-defying quadruped, also presented a claim, accompanied with a bill of particulars. At this juncture, a fellow appeared, offering to swap his eleven-year-old-fresh-milch-cow, of steady and matronly habits, warranted to give twelve quarts of milk at a "sitting," and do it every day the season through, for five dollars to boot. This was an opportunity not to be lost. So, we swapped, "unsight and unseen." Would you believe it? It required nearly twelve days for that blamed old cow to give *one* quart of milk! Now, what sort of a duplex-spotter-bell-punch did *that* fellow need?

---

FOGS.—There is an opinion prevalent generally that fogs are indicators of the existence of malaria in the districts where they occur, which is entirely incorrect. Webster's definition of "fog" tells the whole story in as few words as possible, *i. e.*, "Watery vapor, precipitated in the lower part of the atmosphere and disturbing the transparency. It differs from cloud only in being near the ground." Under all circumstances there is from the earth's surface constantly an evaporation which has been estimated by scientists who have experimented at different hours of day and night and in different temperatures of earth and atmosphere to amount to about one hundred thousand cubic miles of water raised into the atmosphere every year. This, after coming in contact at certain heights with the cooler strata, is condensed into clouds and again returns to the earth in the form of rain, thus keeping up the equilibrium, which would soon be destroyed if the evaporation and condensation and return did not keep pace. Now this process is going on constantly to a greater or less degree, according to the circumstances of heat and cold, excessive rain or drought, etc., in the special locality, yet it is only made apparent to us when from any sudden change in temperature the invisible vapor becomes chilled at the earth's surface instead of in the rarified and colder upper air. When it becomes a cloud on the earth instead of a cloud high above the earth's surface, the cloud on the surface is simply a fog and as soon as the equilibrium of temperature is restored between the earth and the lower stratum of atmosphere continues its journey upwards until it meets another cold stratum and is again condensed into fog, but under the accepted term of "cloud." From this explanation of "Fogs" it can readily be seen that the changes of temperature that produce them may exist and do exist in the healthiest regions, that they are only the condensation of what is constantly present to us and are not necessarily charged with any *mala aria* (bad air) or any specific poison of telluric or vegetable origin known under the title of malaria or miasma.

We are not disposed to argue that when miasma or malaria does exist in any locality, that during exposure to a fog, with a predisposition to ague a person might not be more liable to succumb to that influence, because the sudden changes which produce fog also affect the human system and also, perhaps, the condensation of the unhealthy exhalations may render them more dangerous.

During the severe and continuous fogs in London of 1879–80, in which the absence of high winds and other natural sanitary agents left the impurities in the atmosphere of a great city unremoved, the death rate was greatly increased. This was not the case in other parts of Great Britain where the fogs prevailed.

We claim, then, that fogs are not to be feared as of malarial origin, nor are they any more deleterious to health than sudden changes of temperature under any condition.

Indeed, we may find them very useful as actual indicators of changes which we should note and might neglect if the cloud were not present. Many fields of tobacco and corn have been saved from the early frosts of this year by the warm, damp blanket of fog nature threw over them.

---

THE GLORIOUS MINORITY.—We Americans are so accustomed to the rule of the majority that we have come to accept it as a sort of Divine Right, which it is almost blasphemous to question. "*Vox Populi, Vox Dei,*' "The voice of the People (with a big P) is the voice of God," is the popular shibboleth, and a denial of this faith, or even a half-hearted support of it, would be enough to ruin the prospects of any "rising politician." But, suppose we drop the "Bird o' Freedom" business, and look at the matter calmly and dispassionately, without fear or favor—shall we find that "The voice of the People *is* the voice of God," and that the majority *ought* to rule because it is more likely to be right, or that the reverse is true, and that the rule of the majority is "government by the most ignorant because there happens to be most of them?"

In every community there are a few people who know more than the mass of their fellow-citizens, and the number of those who are at once good, wise and true is not so large as those who possess these desirable qualifications in a lesser degree.

Noah had an overwhelming majority against him on the ark question; Lot would have been defeated for mayor of Sodom by an unprecedented majority; Pontius Pilate permitted the sentence of crucifixion to be passed upon Jesus in obedience to the dictates of a majority of the people of Jerusalem, notwithstanding that he himself was obliged to declare, "I find no fault in Him;" Copernicus was in the minority on the solar system; Galileo on the question of the earth's rotation—but in all these cases the minority was *right*, and the majority was *wrong*. It will not do to come down much closer to our own times for instances, lest we lay ourself open to the charge of partisanship; but the fact that any idea or opinion is held by the minority is *prima facie* evidence of its correctness—that is, if it is a *new* idea. With old ideas it is different, for thick-headed as the majority necessarily is, it is not absolutely proof against the introduction of correct ideas, if they can be forced upon it with sufficient persistency for a sufficient length of time; but it does take time, and meanwhile the "Glorious Minority" have gone forward to a still higher plane, and are as far above and beyond the majority as ever.

Why, if one wanted to test the probable truth of any new statement in science, it would be safe enough to tell it to the first ten people who would listen to it, and if six or more accepted it to lay it away as a probable fallacy.

The majority are usually wrong, and they accept a new humbug with an eagerness proportioned to its absurdity, and reject a new fact in science with an incredulity which varies inversely to its inherent probability. That statement too strong? No, we think not. Look at the instantaneous popular acceptance of the Blue Glass cure. The central idea that the whole was greater than a part (the blue ray alone than the entire spectrum) was a self-evident absurdity, and the thermo-electric theory by which asserted virtues of blue glass were sought to be explained was equally ridiculous, but the *majority* believed it with great enthusiasm and a unanimity very much the same as that with which it *refused* to believe in the locomotive, the telegraph and the telephone until these things were forced upon its acceptance by the cold logic of physical success.

No, we don't want to be with the majority—we don't want to be in the wrong. "For wide is the gate and broad is the way and many there be that go in thereat." If we can have our choice we want to be one of that select company, "The Glorious Minority," every time. When old Harry Clay said, "I'd rather be *right* than be President," he said a noble thing, worth all the buncombe about "all men being created free and equal," &c., &c., that that prince of demagogues, Mr. Thos. Jefferson, ever wrote; but at the same time he passed a severe criticism on the goodness and good sense of the majority by showing how inherently improbable he felt it to be that under the rule of the majority he could ever be *both* right and President at the same time.

It is the "Glorious Minority" that has led the world from darkness to light. Condemned, despised, rejected during life, they have triumphed in the end; while the dull majority, rejecting, rebelling, resisting the "Glorious Minority" of its own time, eagerly seizes upon the cast off ideas of the minority of a preceding age, and thus furnished forthwith proceeds to combat, and, if possible, to crush the ideas which it will end by accepting when they, too, have become old and worn. Meanwhile this burly giant, whose sole merit is his bigness, boasts himself, like him of Gath, and proclaims himself a god. "*Vox populi, vox dei!*" "Hear my brazen trumpet—*I* blow it!" And so offends high heaven till the pebble from the sling of the shepherd lad records another triumph for "The Glorious Minority."

---

THE INDEX—for the past three years will be issued with the next number of THE BISTOURY. We will then have a volume of over three hundred pages, fully indexed, and so made valuable for reference upon a multitude of subjects.

